# Prognostic value of NT-proBNP in patients with severe COVID-19

**DOI:** 10.1186/s12931-020-01352-w

**Published:** 2020-04-15

**Authors:** Lei Gao, Dan Jiang, Xue-song Wen, Xiao-cheng Cheng, Min Sun, Bin He, Lin-na You, Peng Lei, Xiao-wei Tan, Shu Qin, Guo-qiang Cai, Dong-ying Zhang

**Affiliations:** 1 Department of Cardiovascular Medicine, The First Affiliated Hospital of Chongqing Medical University, Chongqing, 400016 China; 2 Department of Cardiovascular Medicine, The First Branch of the First Affiliated Hospital of Chongqing Medical University, Chongqing, 400016 China; 3 Traditional Chinese Medicine hospital Dianjiang Chongqing, Chongqing, 408300 China

**Keywords:** NT-proBNT, COVID-19, SARS-CoV-2, Outcome

## Abstract

**Background:**

The outbreak of coronavirus disease 2019 (COVID-19) caused by severe acute respiratory syndrome coronavirus 2 (SARS-CoV-2) in China has been declared a public health emergency of international concern. The cardiac injury is a common condition among the hospitalized patients with COVID-19. However, whether N terminal pro B type natriuretic peptide (NT-proBNP) predicted outcome of severe COVID-19 patients was unknown.

**Methods:**

The study initially enrolled 102 patients with severe COVID-19 from a continuous sample. After screening out the ineligible cases, 54 patients were analyzed in this study. The primary outcome was in-hospital death defined as the case fatality rate. Research information and following-up data were obtained from their medical records.

**Results:**

The best cut-off value of NT-proBNP for predicting in-hospital death was 88.64 pg/mL with the sensitivity for 100% and the specificity for 66.67%. Patients with high NT-proBNP values (> 88.64 pg/mL) had a significantly increased risk of death during the days of following-up compared with those with low values (≤88.64 pg/mL). After adjustment for potential risk factors, NT-proBNP was independently correlated with in-hospital death.

**Conclusion:**

NT-proBNP might be an independent risk factor for in-hospital death in patients with severe COVID-19.

**Trial registration:**

ClinicalTrials, NCT04292964. Registered 03 March 2020,

## Background

The outbreak of coronavirus disease 2019 (COVID-19) caused by severe acute respiratory syndrome coronavirus 2 (SARS-CoV-2) in China has been declared a public health emergency of international concern on 30 January 2020 [1]. Despite lower case fatality rate, SARS-CoV-2 has killed more people than SARS and MERS and the number keeps growing [2]. Epidemic studies have described that patients with severe COVID-19 were more likely to develop adverse clinical outcomes with more complications including acute respiratory distress syndrome, acute cardiac injury, acute kidney injury and shock [3, 4]. Investigating prognostic markers for severe patients provides insights for early therapeutic strategies.

Cardiac injury is a common condition among the hospitalized patients with COVID-19. It was recently reported that 19.7% patients from a total of 416 cases with COVID-19 had cardiac injury with more adverse clinical outcomes compared to those without cardiac injury [5]. Guo et al. also reported that COVID-19 patients with elevated TnT levels had higher mortality [6]. A retrospective, single-center case series of the 138 COVID-19 patients study reported that 7.2 and 16.7% patients had complications of acute cardiac injury and arrhythmia, respectively [7]. The fraction of acute cardiac injury and arrhythmia was even higher in severe patients with the percentage of 22.2 and 44.4%, respectively. The patients with severe COVID-19 also showed higher creatine kinase-MB (CK-MB) and hypersensitive troponin I (hs-TnI) levels than others [7].

A recent study demonstrated that the heart failure marker, N terminal pro B type natriuretic peptide (NT-proBNP), increased significantly during the course of hospitalization in those who ultimately died [6]. However, there is no research concerning whether NT-proBNP predicted the outcome of severe COVID-19 patients.

## Methods

### Subjects

The study initially enrolled 102 patients with severe COVID-19 from a continuous sample in Hubei General Hospital during the management by national medical team. The study is a retrospective, observational registry with clinicaltrials.gov identifier NCT04292964. The study was also registered on Chinese medical research registration information system. All procedures were followed the instructions of local ethic committee (approval NO. 20200701). Criteria for severe conditions included respiratory rate ≥ 30/min or rest oxyhemoglobin saturation (SPO_2_) ≤93% or oxygenation index (arterial oxygen tension/ inspired oxygen fraction, PaO2/FiO2) ≤300 mmHg. All the data was collected using a same protocol by well-trained researchers with a double-blind method. Patients lacking NT-proBNP results (*n* = 45) were excluded. Patients who had stroke (*n* = 2) and acute myocardial infarction (*n* = 1) were excluded. Other exclusion criteria including patients with malignant tumor (*n* = 0) and pregnancy (*n* = 0) were also taken account. Finally, 54 patients with COVID-19 were studied in this research.

### Baseline data and follow-up

Demographic data, clinical features and medical history were available and collected according to the patient record system. Data collection of laboratory results were defined using the first-time examination at admission (within 24 h after admission). All the laboratory data was tested in a same laboratory with the same standard. To observe the risk of in-hospital death, patients were followed up from admission to discharge (1 to 15 days). The primary outcome was in-hospital death defined as the case fatality rate. The follow-up data were collected from reviewing medical records by trained researchers using double-blind method.

### Statistical analysis

Data is presented as mean ± standard deviation, frequency (%) or median (interquartile ranges). Intergroup comparisons between NT-proBNP high group and low group were made by the independent-samples *T*-test (normally distributed continuous variables), Mann-Whitney *U* test (nonnormally distributed continuous variables) and chi-square test (categorical variables). The best NT-proBNP cut-off was that of the highest product of sensitivity and specificity for in-hospital death prediction. Cumulative survival curves of in-hospital death were estimated using the Kaplan-Meier product-limit estimation method with the log-rank test. Spearman correlation analysis was used to investigate the coefficients of NT-proBNP with selected covariates. Cox proportional hazards models were used to screening out the potential risk factors and analyzing the independent effect of NT-proBNP for in-hospital death. Statistical analyses were performed by SPSS 22.0 (SPSS, Chicago, IL, USA) and a two-sided *P* < 0.05 was considered statistically significant.

## Results

### Baseline characteristics

**Fig. 1:**
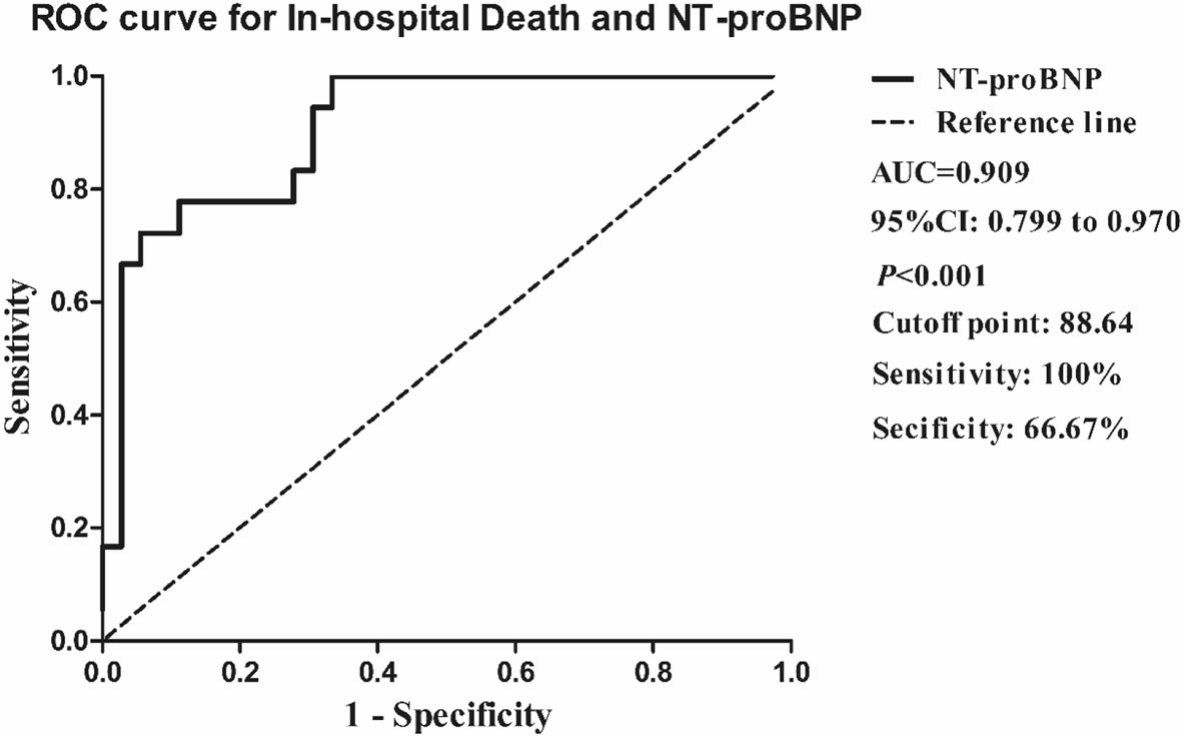
The NT-proBNP for in-hospital death of coronavirus disease 2019 (COVID-19) by receiver operating characteristic (ROC) curves. The area under the curve (AUC) of NT-proBNP was 0.909. The best cutoff of NT-proBNP for prediction in-hospital death was 88.64 pg/mL with the sensitivity of 100% and the specificity of 66.67%. 95% CI, 95% confidence interval

Baseline characteristics of participants were divided into two groups by low and high NT-proBNP levels (NT-proBNP≤88.64 pg/mL and NT-proBNP> 88.64 pg/mL, Table 1) according to the cut-off value determined in the ROC curve (Fig. 1). Patients in NT-proBNP high group were significantly older with more comorbidities of hypertension (HP) and coronary heart disease (CHD), higher levels of diastolic blood pressure (DBP), myohemoglobin (MYO), CK-MB, hs-TnI, blood urea, creatinine, white blood cell (WBC), C-Reactive Protein (CRP) and procalcitonin (PCT) and lower level of lymphocyte (LYM) than the participants in NT-proBNP low group. Other characteristics like sex, temperature, pulse rate, respiratory rate, systolic blood pressure and the history of chronic obstructive pulmonary disease (COPD) and diabetes showed no significance between two groups with the different levels of NT-proBNP (Table 1.).

**Table 1:** Baseline characteristics of total and different degrees of NT-proBNP

Characteristics	Total (*n* = 54)	NT-proBNP≤88.64 pg/ml (*n* = 24)	NT-proBNP> 88.64 pg/ml (*n* = 30)	*P*
Male/Female (n)	24/30	8/16	16/14	0.142
Age (years)	60.4 ± 16.1	51.6 ± 13.9	67.4 ± 14.4	< 0.001
Temperature (°C)	36.7 (36.5–36.9)	36.8 (36.5–36.9)	36.6 (36.5–36.9)	0.670
Pulse (/min)	82 (76–97)	84 (76–97)	82 (76–96)	0.679
Respire (/min)	20 (19–21)	20 (19–20)	20 (18–26)	0.209
SBP (mmHg)	128 (119–138)	126 (115–134)	129 (120–144)	0.218
DBP (mmHg)	78 (70–83)	73 (69–78)	80 (70–86)	0.040
History of HP (n)	12 (22.2%)	2 (8.3%)	10 (33.3%)	0.028
History of CHD (n)	9 (16.7%)	1 (4.2%)	8 (26.7%)	0.027
History of COPD (n)	2 (3.7%)	0	2 (6.7%)	0.197
History of DM (n)	8 (14.8%)	3 (12.5%)	5 (16.7%)	0.668
NT-proBNP (pg/ml)	137.30 (39.64–494.98)	37.28 (22.28–61.74)	420.40 (199.63–919.88)	< 0.001
MYO (ng/ml)	39.28 (26.26–86.84)	25.35 (14.04–35.20)	82.53 (34.55–123.96)	< 0.001
CK-MB (ug/L)	1.04 (0.65–2.27)	0.63 (0.37–0.79)	1.90 (1.08–3.78)	< 0.001
Hs-TnI (ng/ml)	< 0.006 (< 0.006–0.022)	< 0.006 (< 0.006- < 0.006)	0.021 (< 0.006–0.136)	0.001
Urea (mmol/L)	4.8 (3.3–9.0)	3.4 (2.6–4.9)	7.1 (4.4–9.9)	< 0.001
Creatinine (umol/L)	63 (44–77)	54 (41–69)	79 (55–86)	0.016
WBC (10^9^/L)	5.42 (4.13–7.45)	5.89 (4.53–10.76)	5.73 (4.50–8.20)	0.007
LYM (10^9^/L)	1.12 ± 0.52	1.30 ± 0.43	0.98 ± 0.55	0.021
CRP (mg/L)	34.8 (5.3–61.0)	7.6 (5.0–34.8)	54.3 (14.3–117.9)	0.003
PCT (ng/ml)	0.063 (0.029–0.171)	0.038 (0.020–0.058)	0.137 (0.049–0.468)	< 0.001
In-hospital death (n)	18 (33.3%)	0	18 (60.0%)	< 0.001

Abbreviations: *SBP* Systolic blood pressure, *DBP* Diastolic blood pressure, *HP* Hypertension, *CHD* Coronary heart disease, *COPD* Chronic obstructive pulmonary disease, *DM* Diabetes mellitus, *NT-proBNP* N-terminal pro-brain natriuretic peptide, *MYO* Myoglobin, *CK-MB* creatine kinase-MB, *Hs-TnI* High-sensitivity troponin-I, *WBC* White blood cell, *LYM* Lymphocytes, *CRP* C-reactive protein, *PCT* Procalcitonin

### Receiver operator characteristic (ROC) curve for prediction in-hospital death

Receiver operation characteristic (ROC) curves were shown in Fig. 1 to analyze the prognostic value and the best cut-off of NT-proBNP for prediction in-hospital death. The area under the curve (AUC) for in-hospital death was 0.909 (95%CI 0.799–0.970, *P* < 0.001). The best cut-off of NT-proBNP for predicting in-hospital death was 88.64 pg/mL with the sensitivity for 100% and the specificity for 66.67% (Fig. 1).

### Cumulative survival curves of in-hospital death

**Fig. 2:**
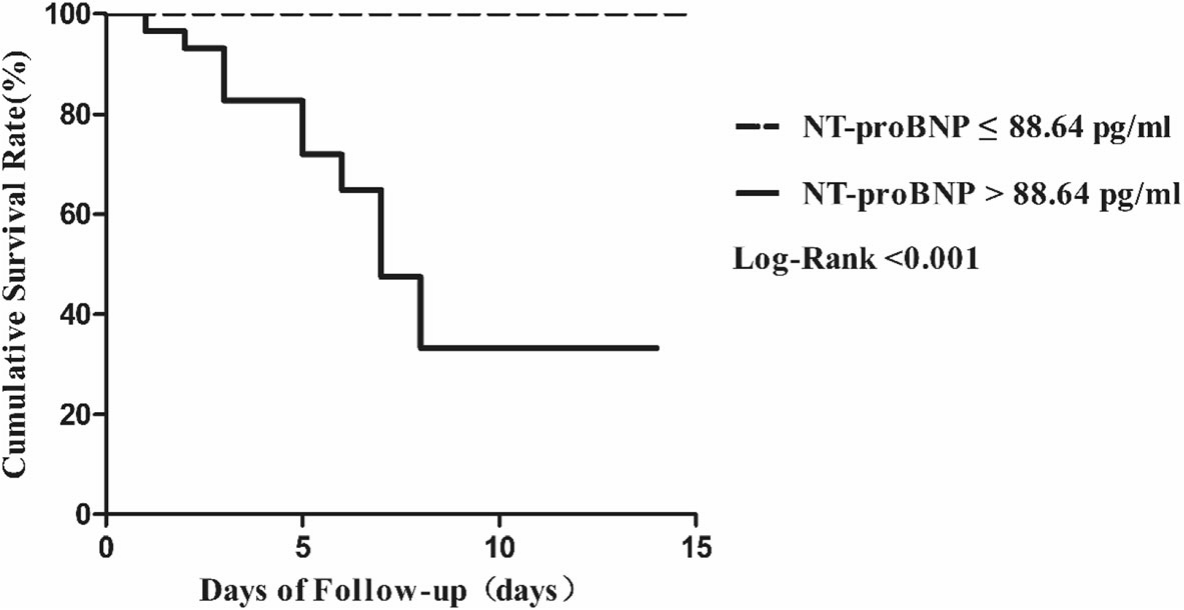
Kaplan-Meier plots showing the cumulative survival rate of COVID-19 patients who were stratified into two groups according to plasma NT-proBNP cutoff point at baseline. Dotted line, NT-proBNP ≤88.64 pg/ml, *n* = 24; Solid line, NT-proBNP > 88.64 pg/ml, *n* = 30; log-rank test for trend, *P* < 0.001)

Cumulative survival rate curves between two groups categorized by NT-proBNP cut-off value were shown in Fig. 2. Patients in high NT-proBNP (> 88.64 pg/mL) group had a significantly higher risk of death during the days of following-up than the low group (NT-proBNP≤88.64 pg/mL) (Fig. 2).

### Spearman correlation coefficients of NT-proBNP with selected covariates

In present study, plasma NT-proBNP was positively correlated with age, urea, cardiac injury markers of MYO, CK-MB and hs-TnI and systematic inflammation makers of WBC, CRP, Hs-CRP and PCT (Supplemental Table. 1).

### Results of cox proportional hazards analyses of in-hospital death

Cox proportional hazards regression analysis was used to evaluate potential associations between NT-proBNP and in-hospital death. Results of univariate analyses showed that the hazard ratio (HR) of NT-proBNP associated to in-hospital death was 1.369 (95% CI 1.217–1.541, *P* < 0.001) for an increase of 100 pg/mL. Meanwhile, age, male, history of hypertension (HP), myoglobin (MYO), creatine kinase-MB (CK-MB), high-sensitivity troponin-I (Hs-TnI), urea, creatinine, white blood cell (WBC), lymphocytes (LYM), c-reactive protein (CRP) and procalcitonin (PCT) were correlated with the risk of in-hospital death (Table 2).

**Table 2:** Results of univariate Cox proportional-hazards regression analyzing the effect of baseline variables on in-hospital death

Characteristics		HR (95%CI)	*P*
Sex	Male	–	
	Female	0.348 (0.130–0.930)	0.035
Age, per 10 years		1.975 (1.309–2.981)	0.001
History of HP	no	–	–
	yes	4.044 (1.604–10.200)	0.003
History of CHD	no	–	–
	yes	2.652 (0.992–7.092)	0.052
History of COPD	no	–	–
	yes	4.127 (0.945–18.024)	0.059
History of DM	no	–	–
	yes	0.958 (0.277–3.314)	0.947
NT-proBNP, per 100 pg/ml		1.369 (1.217–1.541)	< 0.001
MYO, per 1 ng/ml		1.006 (1.003–1.008)	< 0.001
CK-MB, per 1 μg/L		1.259 (1.098–1.443)	0.001
Hs-TnI, per 1 ng/ml		1.862 (1.273–2.722)	0.001
Urea, per 1 mmol/L		1.134 (1.073–1.198)	< 0.001
Creatinine, per 1 umol/L		1.028 (1.013–1.043)	< 0.001
WBC, per 1 × 10^9^/L		1.150 (1.076–1.229)	< 0.001
LYM, per 1 × 10^9^/L		0.065 (0.017–0.249)	< 0.001
CRP, per 1 mg/L		1.021 (1.012–1.030)	< 0.001
PCT, per 0.1 ng/ml		1.241 (1.142–1.349)	< 0.001

Abbreviations: *HP* Hypertension, *CHD* Coronary heart disease, *COPD* Chronic obstructive pulmonary disease, *DM* Diabetes mellitus, *NT-proBNP* N-terminal pro-brain natriuretic peptide, *MYO* Myoglobin, CK-MB Creatine kinase-MB, *Hs-TnI* High-sensitivity troponin-I, *WBC* White blood cell, *LYM* Lymphocytes, *CRP* C-reactive protein, *PCT* Procalcitonin, *HR* hazards ratio, *95%CI* 95% confidence interval

**Fig. 3:**
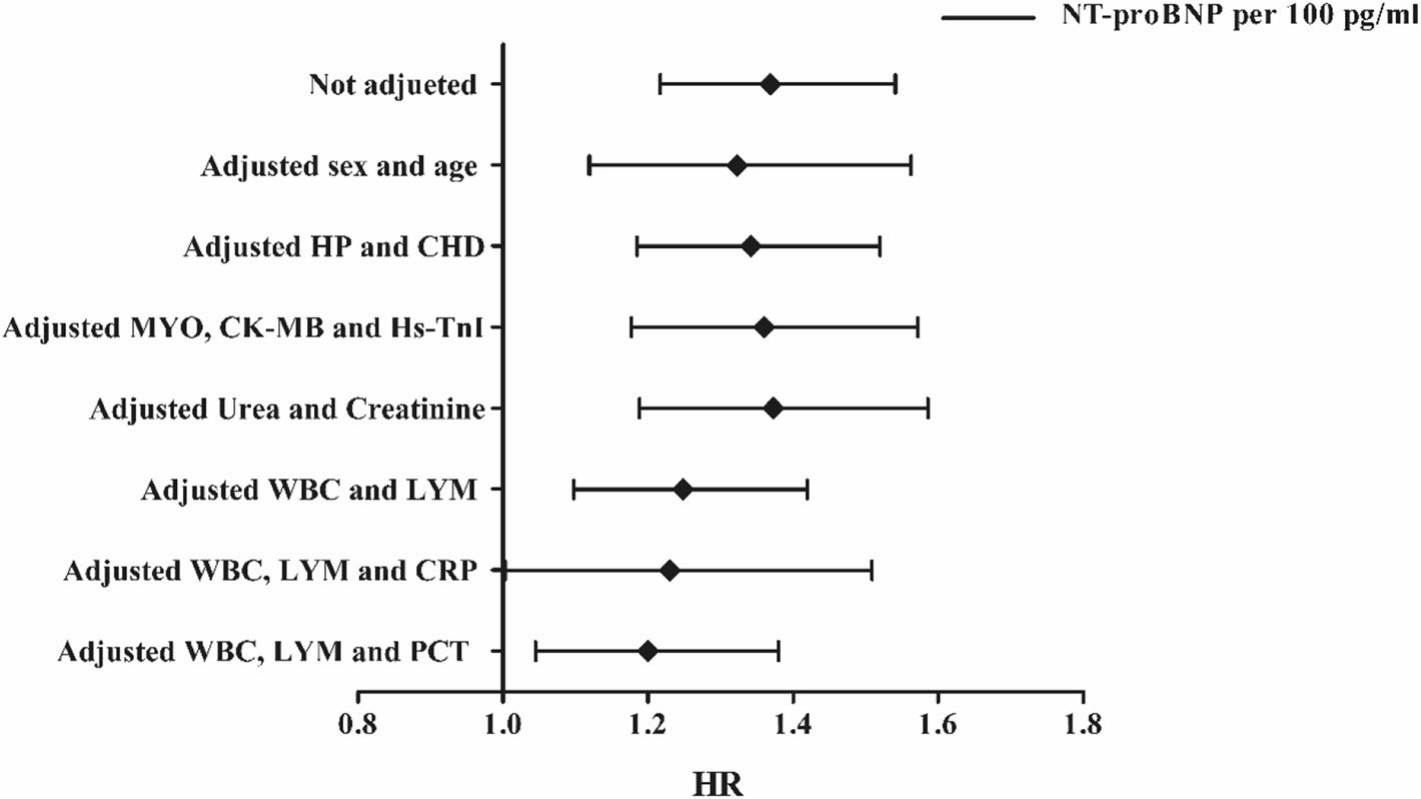
Forest plots of multivariate Cox proportional-hazards regression analyzing the effect of baseline variables on in-hospital death**.** HP, hypertension; CHD, coronary heart disease; MYO, myoglobin; CK-MB, creatine kinase-MB; Hs-TnI, high-sensitivity troponin-I; WBC, white blood cell; LYM, lymphocytes; CRP, c-reactive protein; PCT, procalcitonin; HR, hazards ratio

Multivariate Cox proportional hazards regression analyses were used to evaluate the independent prognostic effect of NT-proBNP level. After adjusting for sex and age (Mode 1), the HR of NT-proBNP for in-hospital death was 1.323 (95% CI 1.119–1.563, *P* = 0.001) for an increase of 100 pg/mL. After adjusting for HP and CHD history (Mode 2), the HR was 1.342 (95% CI 1.185–1.520, *P* < 0.001). After adjusting for MYO, CK-MB and hs-TNI (Mode 3), the HR was 1.360 (95% CI 1.177–1.572, *P* < 0.001). After adjusting for urea and creatinine (Mode 4), the HR was 1.373 (95% CI 1.188–1.586, *P* < 0.001). After adjusting for WBC and LYM (Mode 5), the HR was 1.248 (95% CI 1.097–1.419, *P* = 0.001). After adjusting for WBC, LYM and CRP (Mode 6), the HR was 1.230 (95% CI 1.003–1.509, *P* = 0.047). After adjusting for WBC, LYM and PCT (Mode 7), the HR was 1.200 (95% CI 1.045–1.380, *P* = 0.010). In the process, the HRs of WBC and PCT in Mode 5 and 7 also showed significance for independently predicting in-hospital death while LYM show protective effect (Table 3, Fig. 3).

**Table 3:** Results of multivariate Cox proportional-hazards regression analyzing the effect of baseline variables on in-hospital death

Mode	HR (95%CI)	*P*
Not Adjusted NT-proBNP, per 100 pg/ml	1.369 (1.217–1.541)	< 0.001
**Mode 1**		
NT-proBNP, per 100 pg/ml	1.323 (1.119–1.563)	0.001
Female vs. Male	1.077 (0.330–3.518)	0.902
Age, per 10 years	1.176 (0.719–1.922)	0.518
**Mode 2**		
NT-proBNP, per 100 pg/ml	1.342 (1.185–1.520)	< 0.001
HP, yes vs. no	1.613 (0.591–4.406)	0.351
CHD, yes vs. no	1.219 (0.422–3.521)	0.714
**Mode 3**		
NT-proBNP, per 100 pg/ml	1.360 (1.177–1.572)	< 0.001
MYO, per 1 ng/ml	1.001 (0.996–1.005)	0.773
CK-MB, per 1 μg/L	1.119 (0.905–1.385)	0.299
Hs-TnI, per 0.1 ng/ml	1.031 (0.574–1.855)	0.918
**Mode 4**		
NT-proBNP, per 100 pg/ml	1.373 (1.188–1.586)	< 0.001
Urea, per 1 mmol/L	1.041 (0.936–1.158)	0.460
Creatinine, per 1 umol/L	0.999 (0.974–1.025)	0.957
**Mode 5**		
NT-proBNP, per 100 pg/ml	1.248 (1.097–1.419)	0.001
WBC, per 1 × 10^9^/L	1.099 (1.015–1.190)	0.021
LYM, per 1 × 10^9^/L	0.163 (0.035–0.761)	0.021
**Mode 6**		
NT-proBNP, per 100 pg/ml	1.230 (1.003–1.509)	0.047
WBC, per 1 × 10^9^/L	1.036 (0.903–1.189)	0.611
LYM, per 1 × 10^9^/L	0.201 (0.033–1.221)	0.081
CRP, per 1 mg/L	1.011 (0.999–1.023)	0.066
**Mode 7**		
NT-proBNP, per 100 pg/ml	1.200 (1.045–1.380)	0.010
WBC, per 1 × 10^9^/L	1.088 (0.016–1.164)	0.016
LYM, per 1 × 10^9^/L	0.151 (0.029–0.778)	0.024
PCT, per 0.1 ng/ml	1.110 (1.010–1.220)	0.030

Abbreviations: *NT-proBNP* N-terminal pro-brain natriuretic peptide, *HP* Hypertension, *CHD* Coronary heart disease, *MYO* Myoglobin, *CK-MB* Creatine kinase-MB, *Hs-TnI* High-sensitivity troponin-I, WBC White blood cell, LYM Lymphocytes, CRP C-reactive protein, PCT Procalcitonin, HR Hazards ratio; 95%CI 95% confidence interval

## Discussion

The present study for the first time showed the relationship between plasma NT-proBNP level and the risk of in-hospital death in severe COVID-19 patients. Severe COVID-19 patients with high NT-proBNP levels tended to be older with increased cardiac injury markers and higher levels of systematic inflammation markers. Patients with high NT-proBNP (> 88.64 pg/mL) level had lower cumulative survival rate. After adjusting for potential cofounders in separate modes, NT-proBNP presented as an independent risk factor of in-hospital death in patients with severe COVID-19.

Previous studies have found that NT-proBNP is a powerful and independent predictor of mortality in community-acquired pneumonia (CAP) [8–10]. In these studies, the best cut-off values of NT-proBNP for prediction 30-day mortality were 1434.5 pg/mL and 1795.5 pg/mL, respectively [8, 10]. The elevated NT-proBNP in these cases was believed owing to the cardiac complications resulted from complex interactions among preexisting conditions, relative ischemia, up-regulation of the sympathetic system, systemic inflammation and direct pathogen-mediated damage to the cardiovascular system [11].

However, the cutoff value of NT-proBNP to predict the adverse outcome of severe COVID-19 patients was far lower than the threshold to diagnose heart failure (450 pg/mL for < 50 years, 900 pg/mL for 50–75 years and 1800 pg/mL for > 75 years) [12] in present study. It was suggested that the prognostic effect of plasma NT-proBNP in severe COVID-19 patients could not fully ascribe to heart failure induced by the virus or hypoxia. Further understanding of physiological and pathological significance of plasma NT-proBNP elevation in severe COVID-19 patients might help clinicians make corresponding decisions to reduce the risks of adverse outcome.

NT-proBNP is secreted in response to increased myocardial wall stress [13]. It is also controlled by acute renal injury and proinflammatory molecules such as lipopolysaccharide, interleukin 1, C-reactive protein, and cardiotrophin I, which are independent of ventricular function [14, 15]. It was consisted with the study finding that NT-proBNP level was positively correlated to the makers of cardiac injury, renal injury and systematic inflammation. And these makers also constituted the risks of in-hospital death according to the univariate Cox proportional-hazards regression analysis. However, NT-proBNP was an independent risk factor after accounting these factors in multivariate Cox. The prognostic effect of NT-proBNP might be a specific index of reflecting the overall state of SARS-CoV-2 infection.

The mechanism of SARS-CoV-2-induced cardiac injury was still unclear. From the result of autopsy by Xu and colleagues, a few interstitial mononuclear inflammatory infiltrates were observed in heart biopsy, indicating an inflammation induced cardiac injury [16]. Other factors including the SARS-CoV-2 infection and invasion cardiomyocytes via the binding site of angiotensin-converting enzyme-related carboxypeptidase (ACE2) [17], the pulmonary infection induced inadequate oxygen supply to the myocardium and the influences of cytokine storm syndrome [18–20] might also contribute to the cardiac injury [21]. All these contributes to the elevation of NT-proBNP and risks of poor prognosis in patients with COVID-19.

The virus itself may also elevate the NT-proBNP level in COVID-19 patients. SARS-CoV-2 binds with ACE2, resulting the uncontrolled releasing of angiotensin 2 (ANGII) and restricted synthesis of ANG1–7 [22]. The latter exerts anti-inflammation effect to protect tissue while ANGII plays in an opposite role and facilitates the secretion of NT-proBNP [22–24]. It indicated that NT-proBNP level might associated with the severity of infection thus leading an adverse outcome, which needs further verification.

By investigating the prognostic effect of NT-proBNP level of severe COVID-19 patients at admission, it might be helpful to early identifying patients with poor prognoses. However, this study was limited by sample size and a single test of NT-proBNP at admission. Larger studies with continuous monitoring of NT-proBNP are necessary to further confirm the prognostic effect of NT-proBNP in patients with severe COVID-19.

## Conclusion

In conclusion, NT-proBNP might be an independent risk factor for in-hospital death in patients with severe COVID-19.

## Supplementary information

**Additional file 1: Table S1.** Spearman correlation coefficients of NT-proBNP with selected covariates.

## Data Availability

The raw data required to reproduce these findings cannot be shared at this time as the data also forms part of an ongoing study.

## Abbreviations

COVID-19: Coronavirus disease 2019
SARS-CoV-2: Severe acute respiratory syndrome coronavirus 2
ROC: Receiver operation characteristic
HR: Hazard ratio
CAP: Community-acquired pneumonia
SBP: Systolic blood pressure
DBP: Diastolic blood pressure
HP: Hypertension
CHD: Coronary heart disease
COPD: Chronic obstructive pulmonary disease
DM: Diabetes mellitus
NT-proBNP: N-terminal pro-brain natriuretic peptide
MYO: Myoglobin
CK-MB: Creatine kinase-MB
Hs-TnI: High-sensitivity troponin-I
WBC: White blood cell
LYM: Lymphocytes
CRP: C-reactive protein
PCT: Procalcitonin
ACE2: Angiotensin-converting enzyme-related carboxypeptidase
ANG: Angiotensin
