# Supplementary material for: Prognostic value of NT-proBNP in patients with severe COVID-19
Source: Respir Res. 2020 Apr 15;21:83. doi: 10.1186/s12931-020-01352-w (PMC7156898; doi:10.1186/s12931-020-01352-w)
Supplement: Supplementary file 1 — Additional file 1: Table S1. Spearman correlation coefficients of NT-proBNP with selected covariates. [file 12931_2020_1352_MOESM1_ESM.docx]

| Supplemental Table 1. Spearman correlation coefficients of NT-proBNP with selected covariates. | | |
| --- | --- | --- |
| Characteristics | NT-proBNP (pg/ml) | |
|  | R | *P* |
| Age (years) | 0.630 | <0.001 |
| Temperature (℃) | -0.124 | 0.375 |
| Pulse (/min) | -0.135 | 0.335 |
| Respire (/min) | 0.242 | 0.081 |
| SBP (mmHg) | 0.347 | 0.014 |
| DBP (mmHg) | 0.314 | 0.026 |
| MYO (ng/ml) | 0.751 | <0.001 |
| CK-MB (ug/L) | 0.736 | <0.001 |
| Hs-TnI (ng/ml) | 0.748 | <0.001 |
| Urea (mmol/L) | 0.679 | <0.001 |
| Creatinine (umol/L) | 0.480 | <0.001 |
| WBC (10^9^/L) | 0.445 | 0.001 |
| LYM (10^9^/L) | -0.428 | 0.001 |
| CRP (mg/L) | 0.700 | <0.001 |
| PCT (ng/ml) | 0.615 | <0.001 |
| NT-proBNP, N-terminal pro-brain natriuretic peptide; SBP, systolic blood pressure; DBP, diastolic blood pressure; MYO, myoglobin; CK-MB, creatine kinase-MB; Hs-TnI, high-sensitivity troponin-I; WBC, white blood cell; LYM, lymphocytes; CRP, C-reactive protein; PCT, procalcitonin. | | |
